# Point-of-care diagnostic assay for the detection of Zika virus using the recombinase polymerase amplification method

**DOI:** 10.1099/jgv.0.001083

**Published:** 2018-06-13

**Authors:** Nadina I. Vasileva Wand, Laura C. Bonney, Robert J. Watson, Victoria Graham, Roger Hewson

**Affiliations:** Public Health England, National Infection Service, Microbiology Services Division, Porton Down, Salisbury, Wiltshire, SP4 0JG, UK

**Keywords:** Zika, RPA, recombinase polymerase amplification, isothermal, point-of-care, field-diagnostic, molecular detection

## Abstract

The sudden and explosive expansion of Zika virus (ZIKV) from the African continent through Oceania and culminating in the outbreak in South America has highlighted the importance of new rapid point-of-care diagnostic tools for the control and prevention of transmission. ZIKV infection has devastating consequences, such as neurological congenital malformations in infants born to infected mothers and Guillain*–*Barré syndrome in adults. Additionally, its potential for transmission through vector bites, as well as from person to person through blood transfusions and sexual contact, are important considerations for prompt diagnosis. Recombinase polymerase amplification (RPA), an isothermal method, was developed as an alternative field-applicable assay to PCR. Here we report the development of a novel ZIKV real-time reverse transcriptase RPA (RT-RPA) assay capable of detecting a range of different ZIKV strains from a variety of geographical locations. The ZIKV RT-RPA was shown to be highly sensitive, being capable of detecting as few as five copies of target nucleic acid per reaction, and suitable for use with a battery-operated portable device. The ZIKV RT-RPA demonstrated 100 % specificity and 83 % sensitivity in clinical samples. Furthermore, we determined that the ZIKV RT-RPA is a versatile assay that can be applied to crude samples, such as saliva and serum, and can be used as a vector surveillance tool on crude mosquito homogenates. Therefore, the developed ZIKV RT-RPA is a useful diagnostic tool that can be transferred to a resource-limited location, eliminating the need for a specialized and sophisticated laboratory environment and highly trained staff.

## Introduction

Zika virus (ZIKV) is a positive-sense single-stranded RNA virus, a member of the genus *flavivirus*, family *Flaviviridae*, with a genome of ~10.2 kb. It is divided into two known lineages, namely the African and Asian [1]. It was initially isolated in 1947 from a sentinel rhesus macaque exposed to the virus in the Ziika Forest in Uganda, and the first human case was reported in 1962 [2, 3]. Subsequently only sporadic cases limited to Africa and Asia were described until 2007, when the first major outbreak occurred in Yap State, Micronesia, followed by outbreaks throughout the Pacific in French Polynesia, the Cook Islands, Easter Island and New Caledonia [4–17]. The Asian lineage virus was then introduced to Brazil, possibly in 2013, sparking an explosive epidemic that spread through the Americas and became a global health concern [18–21]. Although in most cases ZIKV infection manifests with sub-clinical or mild influenza-like symptoms, severe outcomes such as Guillain–Barre Syndrome (GBS) in adults and microcephaly and other congenital neurological malformations in infants born to infected mothers have been linked to the virus [22–37]. Currently, there is no available effective treatment or vaccine against ZIKV, and therefore control measures focus on avoidance [38]. ZIKV is an arbovirus, transmitted during haematophagous feeding through the bite of female *Aedes* mosquito species (primarily *Aedes aegypti*), or by sexual transmission [33, 39–41]. Transmission through the transfusion of infected blood or organ transplant is a risk that prompt identification and diagnosis could help to avoid [42]. ZIKV research is developing rapidly and new guidance on prevention, diagnosis and surveillance is constantly being shaped, as new data become available [43]. Other arboviruses, such as those that cause dengue and chikungunya, are also transmitted by *Aedes* mosquitoes. As these have similar clinical manifestations, an accurate diagnosis is important for patient management, relevant medical advice, epidemiological follow-up, contact tracing and vector control operations.

ZIKV laboratory diagnosis presents a number of challenges. The virus has been described as cross-reacting serologically with other flaviviruses, such as dengue virus, West Nile virus and yellow fever virus [15, 44]. Indeed, the early diagnosis of the outbreak in Micronesia was inaccurate – it was misidentified as dengue virus by a rapid immunoglobulin M (IgM) test [15]. However, additional nucleic acid testing of samples confirmed ZIKV and current diagnostic algorithms include both IgM/IgG enzyme-linked immunosorbent assays (ELISA) and viral RNA detection [45, 46].

In recent years, the developmental pursuit of rapid point-of-care diagnostic tools has become a major focus in addressing global health concerns [47–56]. Point-of-care testing would allow patient diagnoses at home, in the field, or at a local healthcare centre, enabling physicians to shift the focus from medicinal to preventative measures. This can have a powerful impact on transmission and clinical consequences, thus addressing the challenges faced when controlling an outbreak. Although the identification of pathogens using a nucleic acid amplification approach has traditionally employed the polymerase chain reaction (PCR), and numerous advances in the field have streamlined this method significantly, recent outbreaks of Ebola virus and ZIKV in West Africa and South America, respectively, have highlighted its shortcomings [48, 57]. Sample preparation for PCR remains elaborate and requires a specialized laboratory environment, since PCR tends to be more susceptible to the inhibitors present in crude biological samples. In addition, the thermal cycling involved in PCR carries a high energy demand, which makes this method less appropriate to a resource-limited setting. Although PCR remains the diagnostic method of choice in developed countries, that may not be the case during outbreaks in developing countries. In those cases, access to professional healthcare, including specialized and suitably equipped diagnostic laboratories, may be limited. To address these issues, a number of new diagnostic methods for the detection of nucleic acid have been developed and are in the process of being improved [49, 55, 56, 58–61].

One of these methods is the recombinase polymerase amplification (RPA) assay, which relies on enzymatic activity at a single optimum temperature between 37 and 42 °C, rather than temperature cycling, to achieve template denaturation, primer binding and amplification [56, 62, 63]. RPA utilizes a recombinase enzyme whose function is to bind to the primers and guide them to their homologous sequences in the double-stranded DNA template. The resulting d-loop, formed by the displaced DNA strand, is stabilized by single-stranded DNA-binding (SSB) proteins and amplification is then initiated by a polymerase from the primer-binding site. RPA can routinely generate results within 20 min, and often within 3–10 min. The basic RPA can be augmented with a reverse transcriptase enzyme and a fluorescent probe, which allow the detection of an RNA template, as well as DNA, in real time. RPA may also be more tolerant of crude sample material than PCR, eliminating the necessity for a complicated sample extraction procedure. With its ability to proceed at a constant, relatively low temperature, and its simple detection method, the RPA can be performed using a relatively basic portable battery-operated device that can be taken to a field environment. It has the potential to deliver rapid diagnostic results that facilitate quick clinical decisions in an outbreak, such as the recent ZIKV outbreak in South America.

Here we describe the development and validation of a rapid point-of-care RPA assay for the detection of ZIKV nucleic acid as a diagnostic and surveillance tool. It could be applied to an outbreak in a limited resource setting or to monitor infected vector populations in the field.

## Results

### ZIKV RT-RPA target region is highly conserved in a range of ZIKV strains

**Fig. 1.**
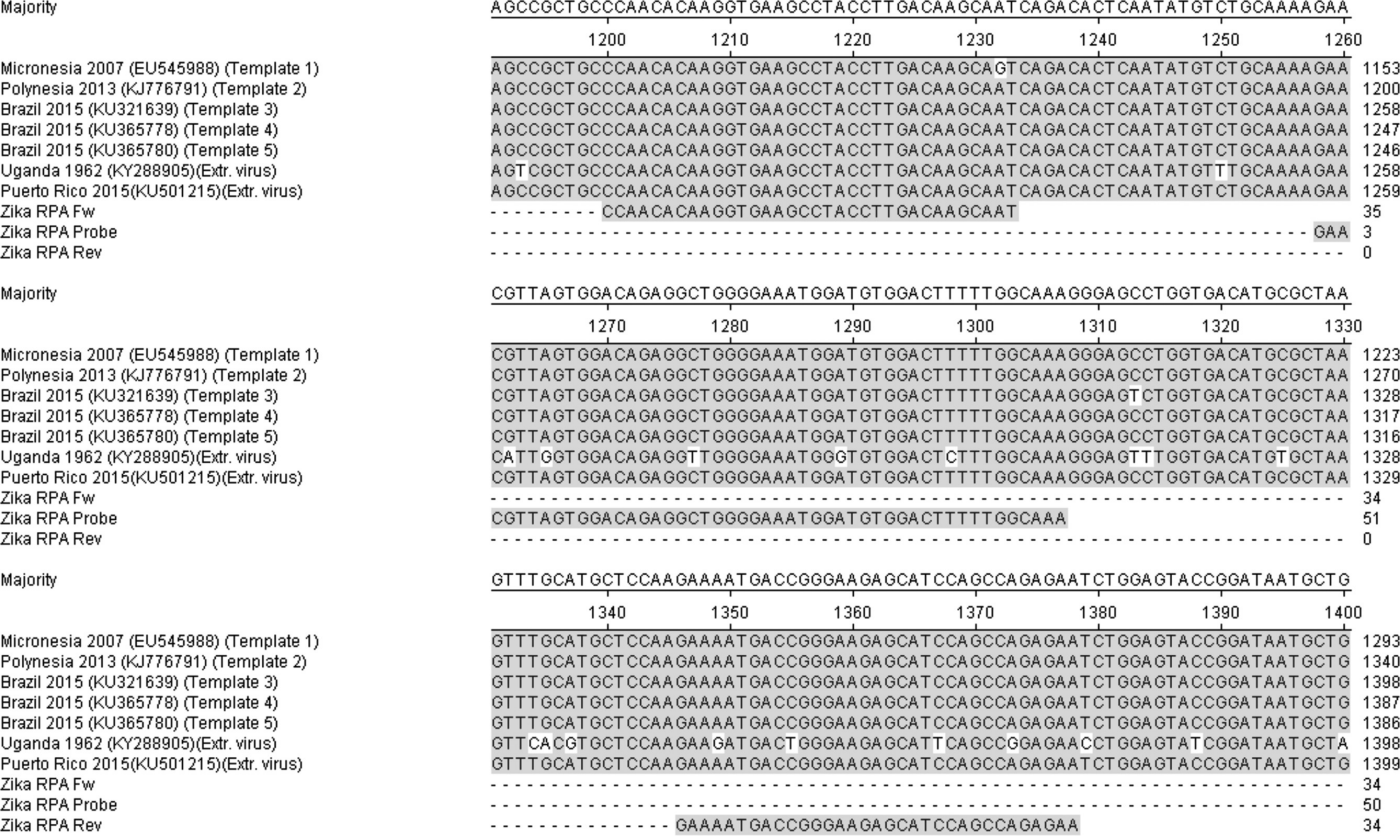
Alignment of synthetic RNA template and extracted cultured Zika virus with the ZIKV RT-RPA primers and probe indicating the RPA target region.

The template region amplified in the ZIKV RT-RPA was selected based on its high level of sequence conservation, as determined by the sequence alignment of strains isolated from different outbreaks (Fig. 1). Asian lineage strains ZIKV 2007 EC (NCBI accession number EU545988), H/PF/2013 (NCBI accession number KJ776791), SPH2015 (NCBI accession number KU321639), BeH819015 (NCBI accession number KU365778), BeH815744 (NCBI accession number KU365780) and PRVABC59 (NCBI accession number KU501215) share a high level of sequence identity over the ZIKV RT-RPA target region (Table 1) [64–67]. This is due to these strains having been isolated more recently, from 2007 until 2015, despite the fact that their locations range from Micronesia and Polynesia to South America (Fig. 1). In contrast, the strain MP1751 (NCBI accession number KY288905) originated from Uganda, Africa, in 1962, and its temporal and geographical distance from the rest of the ZIKV strains is reflected in the greater sequence variation of its ZIKV RT-RPA target region, with the reverse primer differing by three base pairs and the probe by five base pairs from the MP1751 ZIKV strain target region (Table 1) [68]. However, as both the probe and the primers utilized in the RPA assay are longer than those used in a traditional PCR, i.e. 33–50 bp in the case of the ZIKV RT-RPA assay, we expected that this would provide a sufficient footprint to accommodate a certain level of sequence variation and allow for the target to be successfully detected. Additionally, blast searches of the primers and probe, as well as the target region, only identified ZIKV sequences, lending confidence to the specificity of our RPA assay.

**Table 1.** ZIKV RT-RPA assay design A list of sequences employed in the development and validation of the ZIKV RT-RPA assay, including the primers and probe sequences and positions in relation to strain BeH815744 (KU365780) from Brazil, 2015, as well as Zika viral templates (synthetic RNA fragments and extracted cultured virus).

**Sequence ID**	**Description**	**Sequence (5′→3′)**	**Strain and position**
Zika RPA Fw	Zika RPA forward primer	CCAACACAAGGTGAAGCCTACCTTGACAAGCAAT	1186 bp → 1219 bp in KU365780
Zika RPA Rev	Zika RPA reverse primer	TTCTCTGGCTGGATGCTCTTCCCGGTCATTTTC	1332 bp → 1364 bp in KU365780
Zika RPA Probe	Zika RPA exo probe	GAACGTTAGTGGACAGAGGCTGGGGAAATGGA-(Fluorescein-dT)-(d-Spacer)-(BHQ1-dT)-GGACTTTTTGGCAAA-(propanol)	1244 bp → 1293 bp in KU365780
Template 1	1.8 kb RNA fragment from envelope protein E	See Supplementary data (S1)	Strain ZIKV 2007 EC (EU545988) from Micronesia, 2007
Template 2	1.8 kb RNA fragment from envelope protein E	See Supplementary data (S1)	Strain H/PF/2013 (KJ776791) from Polynesia, 2013
Template 3	1.8 kb RNA fragment from envelope protein E	See Supplementary data (S1)	Strain SPH2015 (KU321639) from Brazil, 2015
Template 4	1.8 kb RNA fragment from envelope protein E	See Supplementary data (S1)	Strain BeH819015 (KU365778) from Brazil, 2015
Template 5	1.8 kb RNA fragment from envelope protein E	See Supplementary data (S1)	Strain BeH815744 (KU365780) from Brazil, 2015
African Zika virus	Extracted viral nucleic acid from cultured virus	See Supplementary data (S1)	Strain MP1751 (KY288905) from Uganda, 1962
South American Zika virus	Extracted viral nucleic acid from cultured virus	See Supplementary data (S1)	Strain PRVABC59 (KU501215) from Puerto Rico, 2015

### ZIKV RT-RPA assay can detect as few as five copies of target RNA

To investigate the sensitivity of the ZIKV RT-RPA assay, synthetic RNA fragments representing five different ZIKV virus strains were prepared (Figs 1 and S1, available in the online version of this article). Synthesizing the 1.8 kb RNA fragments allowed for a more accurate quantification of their copy number and a greater precision in determining the limit of detection of the ZIKV RT-RPA assay than could have been obtained using extracted viral nucleic acid. A 10-fold dilution series of the synthetic RNA fragment 5 (strain BeH815744, KU365780, from Brazil, 2015), representing the latest outbreak strain, was prepared and each dilution was tested in the ZIKV RT-RPA assay.

**Fig. 2.**
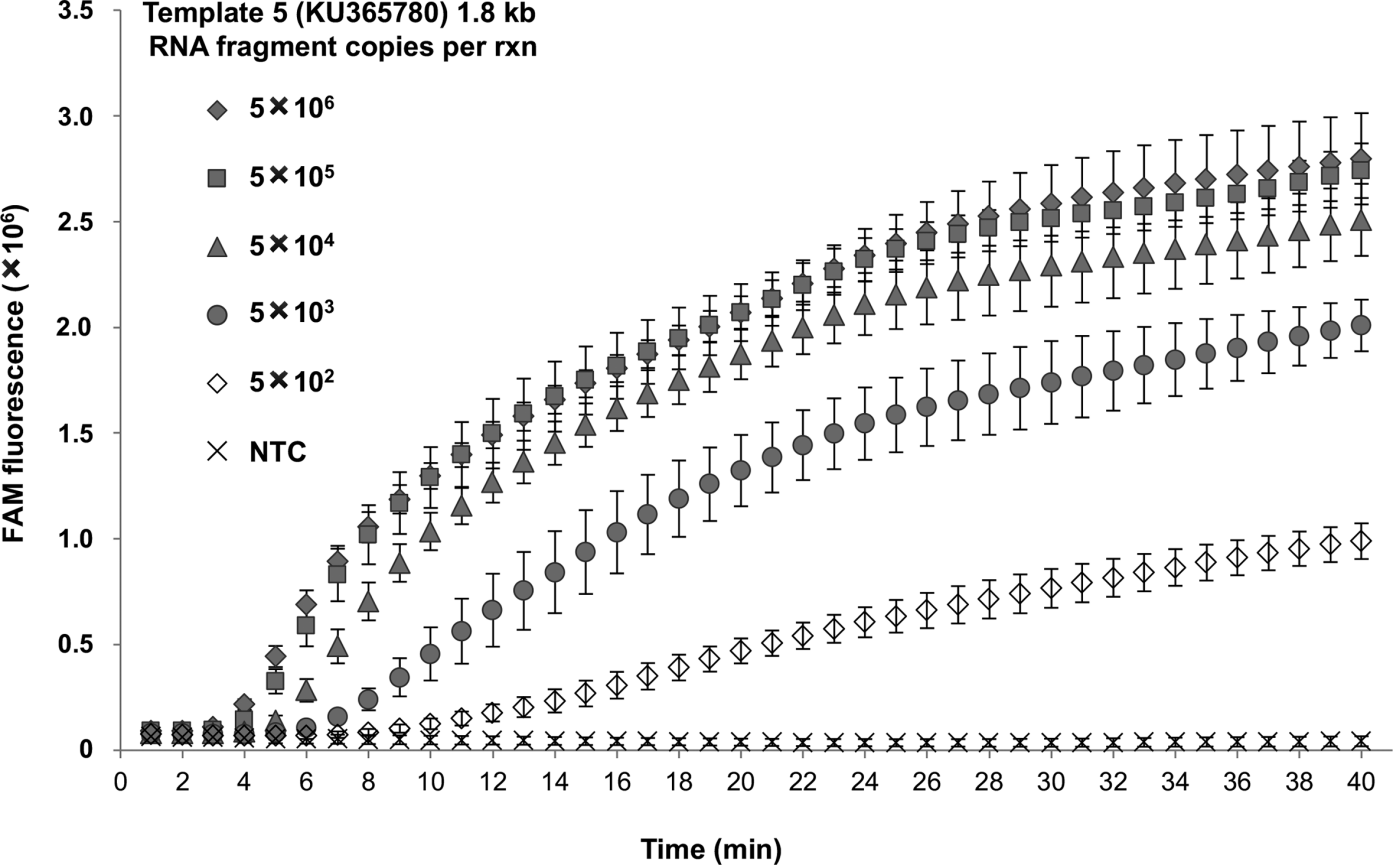
ZIKV RT-RPA assay sensitivity and performance in comparison to ZIKV RT-PCR. Tenfold dilution series of Zika synthetic RNA template 5 (KU365780) used to determine the lowest number of target molecules detected by the ZIKV RT-RPA assay and compared to the background fluorescent signal generated from the non-template control (NTC). Amplification curves show the average total fluorescence values from five independent ZIKV RT-RPA assays; standard deviations are represented as error bars.

It was determined that the ZIKV RT-RPA assay could reliably and rapidly detect 500 copies of the synthetic fragment within 10.72 min on average, which is significantly faster than could be obtained using a conventional real-time PCR assay (Fig. 2, Table 2) [14]. Remarkably, as few as five copies of synthetic template were detected in 22.20 min by the RPA assay in three out of five independent repeats (Fig. S3; Table S1), whereas these samples were not detected by the RT-PCR. The rapidity of the ZIKV RT-RPA assay compared with RT-PCR was demonstrated with 5×10^6^ copies of the synthetic fragment, where our RPA assay gave positive results within 3.38 min (Table 2), whereas RT-PCR required 40.12 min.

**Table 2.** Time to positive (TTP) signal in the detection of different amounts of ZIKV synthetic RNA template 5 (from 5×10^6^ to 5×10^2^ copies per reaction) using the ZIKV RT-RPA assay For comparison, the time necessary to detect the target using the published Zika RT-PCR is also shown.

**Template 5 (KU365780) copies/rxn**	**RT-RPA TTP (min)**	**RT-RPA result**	**RT-PCR TTP (min)**	**RT-PCR result**
5×10^6^	3.38	+ (5/5)	40.12	+ (3/3)
5×10^5^	3.84	+ (5/5)	45.07	+ (3/3)
5×10^4^	4.76	+ (5/5)	49.18	+ (3/3)
5×10^3^	6.48	+ (5/5)	53.38	+ (3/3)
5×10^2^	10.72	+ (5/5)	57.25	+ (3/3)
NTC	Not detected	− (5/5)	Not detected	− (3/3)

### A range of different ZIKV strains are successfully and specifically detected by the ZIKV RT-RPA assay

**Fig. 3.**
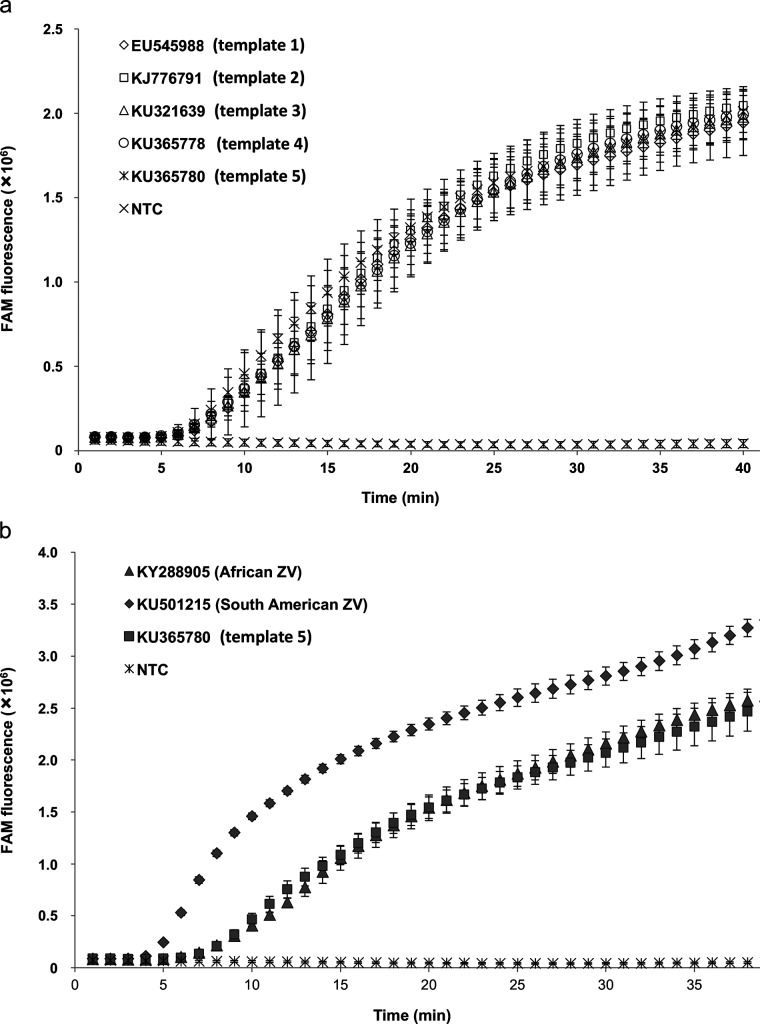
Cross-template detection of the different ZIKV strain targets using the ZIKV RT-RPA. (a) Detection of different ZIKV synthetic RNA templates (1–5) using the ZIKV RT-RPA assay. All synthetic fragments were used at 5×10^3^ copies per reaction and compared to the non-template control (NTC). (b) Amplification of target region in the ZIKV RT-RPA assay using extracted nucleic acid from two strains of cultured ZIKV (African – KY288905 and South American – KU501215) compared to the detection of Zika synthetic RNA fragment 5. Both cultured viral strains were used in the assay at 1.5×10^1^ p.f.u. per reaction, whereas the synthetic fragment 5 was used at 5×10^3^ copies per reaction. The fluorescent signal generated by the non-template control (NTC) is also shown for reference. Amplification curves show the average total fluorescence values from three independent ZIKV RT-RPA assays; standard deviations are represented as error bars.

Cross-strain detection using the ZIKV RT-RPA assay was demonstrated using a range of templates, both synthetic 1.8 kb RNA fragments (templates 1–5) and extracted nucleic acid from two cultured ZIKV strains (Figs 1, 3a, b, S1 and S2). It was observed that all of the five synthesized RNA fragments used at 5×10^3^ copies were successfully amplified in the ZIKV RT-RPA assay with similar efficiency (Fig. 3a). The time to positive (TTP) signal for each of the five synthetic templates was between 7.32 and 8.24 min on average, as determined from three independent biological repeats (Table 3). In contrast, when the same fragments were analysed by ZIKV RT-PCR, the amplification of the target ranged from 53.39 to 54.65 min (Table 3). In addition, the detection of extracted ZIKV nucleic acid utilizing the ZIKV RT-RPA was achieved for both the African and South American strains, with the TTP averaging 7.53 min and 4.52 min, respectively (Fig. 3b). RT-PCR analysis of the extracted viral RNA required 49.16 min for the detection of the African strain and 45.95 min for the South American virus (Table 3), emphasizing the faster performance of the RPA assay.

**Table 3.** Time to positive (TTP) signal in the detection different strains of ZIKV, based on both synthetic RNA fragments (1–5) and extracted nucleic acid from cultured virus of two Zika virus strains (African – KY288905 and South American – KU501215); the ZIKV RT-RPA assay is compared to the performance of the published ZIKV RT-PCR assay

**Sequence ID**	**Description**	**RT-RPA TTP (min)**	**RT-RPA result**	**RT-PCR TTP (min)**	**RT-PCR result**
Template 1 (EU545988)	Synthetic RNA	8.24	+ (3/3)	53.75	+
Template 2 (KJ776791)	Synthetic RNA	7.72	+ (3/3)	54.05	+
Template 3 (KU321639)	Synthetic RNA	7.89	+ (3/3)	54.24	+
Template 4 (KU365778)	Synthetic RNA	8.13	+ (3/3)	54.65	+
Template 5 (KU365780)	Synthetic RNA	7.32	+ (3/3)	53.39	+
African Zika virus (KY288905)	Extracted virus	7.53	+ (3/3)	49.16	+
South American Zika virus (KU501215)	Extracted virus	4.52	+ (3/3)	45.95	+

### The ZIKV RT-RPA assay specifically detects ZIKV

The specificity of the ZIKV RT-RPA was determined using extracted nucleic acid from a panel of viruses pertinent to ZIKV, based on genetic relatedness, clinical relevance and co-circulation (Table 4). The ZIKV RT-RPA assay did not detect the tested members of the following genera: *Orthobunyavirus* (La Crosse and Oropouche viruses), *Phlebovirus* (Rift Valley fever virus) and *Alphavirus* (chikungunya, Mayaro and O’nyong’nyong viruses), which are all mosquito-borne arboviruses with similar distribution and clinical presentation to human ZIKV infection and are, therefore, relevant to the differential diagnosis (Table 4). Other flaviviruses closely related to ZIKV (i.e. dengue 1–4, West Nile, yellow fever, St Louis encephalitis, Powassan, Usutu and Karshi viruses) were also undetected by the ZIKV RT-RPA assay (Table 4). Spondweni virus, belonging to the Spondweni serogroup, to which ZIKV belongs, was also tested using the RT-RPA assay. A highly concentrated sample of Spondweni extracted viral RNA, detected at Ct 22 by a specific real-time PCR, was weakly detected by the ZIKV RT-RPA assay (Fig. S4). However, a 10-fold dilution of the sample abolished the positive signal for Spondweni virus, indicating that in a clinical sample Spondweni viral infection is unlikely to be misidentified as ZIKV (Fig. S4), since viral nucleic acid is less concentrated in clinical samples than in cultured viral RNA extracts.

**Table 4.** Reactivities of flaviviruses, alphaviruses and bunyaviruses in the ZIKV RT-RPA assay Extracted viral RNA from the listed viruses was tested using the ZIKV RT-RPA assay to confirm the specificity of the designed assay for ZIKV.

**Family**	**Virus name**	**Strain**	**RT-RPA result**
*Flaviviridae*	Dengue 1	Hawaii A	Not detected
	Dengue 2	R062	Not detected
	Dengue 3	TC3	Not detected
	Dengue 4	TC25	Not detected
	West Nile	NY99	Not detected
	Yellow fever	FNT	Not detected
	St Louis encephalitis	MSI-7	Not detected
	Powassan	–	Not detected
	Usutu	–	Not detected
	Karshi	30 517	Not detected
	Spondweni	SM-6 V-1s	Partly detected*
*Bunyaviridae*	La Crosse	EVAg stocks, NC_004108	Not detected
	Rift Valley fever	h85/09	Not detected
	Oropouche	EVAg stocks, 005v-EVA832	Not detected
*Alphaviridae*	Chikungunya	–	Not detected
	Mayaro	TC652	Not detected
	O'nyong'nyong	Ang'mom	Not detected

*Spondweni virus, which belongs to the Spondweni serogroup together with ZIKV, was weakly detected at high RNA concentration.

### Extracted ZIKV nucleic acid from clinical samples is successfully and specifically detected by the ZIKV RT-RPA assay

Extracted nucleic acid material was obtained from a selection of 55 clinical samples, including 8 semen, 35 urine, 5 serum, 2 whole blood and 5 other clinical samples, and analysed using the ZIKV RT-RPA assay and compared to RT-PCR assay results (Table 5) 7 were detected as positive by the ZIKV RT-PCR, whereas the remaining 15 were negative using both types of assay. This indicates a sensitivity of 83 % for the ZIKV RT-RPA assay compared to the RT-PCR assay (Table 5)

**Table 5.** Detection of ZIKV nucleic acid using the ZIKV RT-RPA assay in a selection of clinical samples The TTP signal obtained from the ZIKV RT-RPA assay is indicated for a range of patient samples, including semen and urine, and is compared to that generated by the published ZIKV RT-PCR.

**Sample No**	**Sample description**	**RT-RPA TTP (min)**	**RT-RPA result**	**RT-PCR result**
1	Other clinical	13.9	+	+
2	Other clinical	34.8	+	+
3	Other clinical	6.3	+	+
4	Other clinical	15.5	+	+
5	Other clinical	7.2	+	+
6	Semen	37.5	+	+
7	Semen	16.9	+	+
8	Semen	18.2	+	+
9	Semen	Not detected	−	+
10	Semen	6.4	+	+
11	Semen	Not detected	−	+
12	Semen	5.2	+	+
13	Semen	Not detected	−	−
14	Serum	24.8	+	+
15	Serum	37.5	+	+
16	Serum	Not detected	−	−
17	Serum	Not detected	−	−
18	Serum	Not detected	−	−
19	Urine	Not detected	−	+
20	Urine	Not detected	−	−
21	Urine	19.3	+	+
22	Urine	22.5	+	+
23	Urine	34.6	+	+
24	Urine	Not detected	−	−
25	Urine	Not detected	−	−
26	Urine	15.3	+	+
27	Urine	16.2	+	+
28	Urine	Not detected	−	+
29	Urine	33.1	+	+
30	Urine	Not detected	−	−
31	Urine	Not detected	−	−
32	Urine	21.4	+	+
33	Urine	37.5	+	+
34	Urine	35.2	+	+
35	Urine	22.5	+	+
36	Urine	Not detected	−	−
37	Urine	Not detected	−	−
38	Urine	29.5	+	+
39	Urine	36.5	+	+
40	Urine	Not detected	−	−
41	Urine	28.7	+	+
42	Urine	28.8	+	+
43	Urine	Not detected	−	+
44	Urine	Not detected	−	+
45	Urine	Not detected	−	−
46	Urine	24.5	+	+
47	Urine	Not detected	−	−
48	Urine	27.5	+	+
49	Urine	16.3	+	+
50	Urine	Not detected	−	−
51	Urine	Not detected	−	+
52	Urine	19.6	+	+
53	Urine	8.1	+	+
54	Whole blood	20.8	+	+
55	Whole blood	29.0	+	+

In addition, the ZIKV RT-RPA was determined to be 100 % specific, as 11 dengue-positive and 4 chikungunya-positive clinical samples were not detected by the ZIKV RT-RPA (Table S2), and these samples were confirmed to be positive for dengue and chikungunya viral nucleic acid, respectively, by specific published RT-PCR assays [69, 70].

### ZIKV RT-RPA assay may be used as a surveillance tool on crude samples

#### Crude clinical samples

**Fig. 4.**
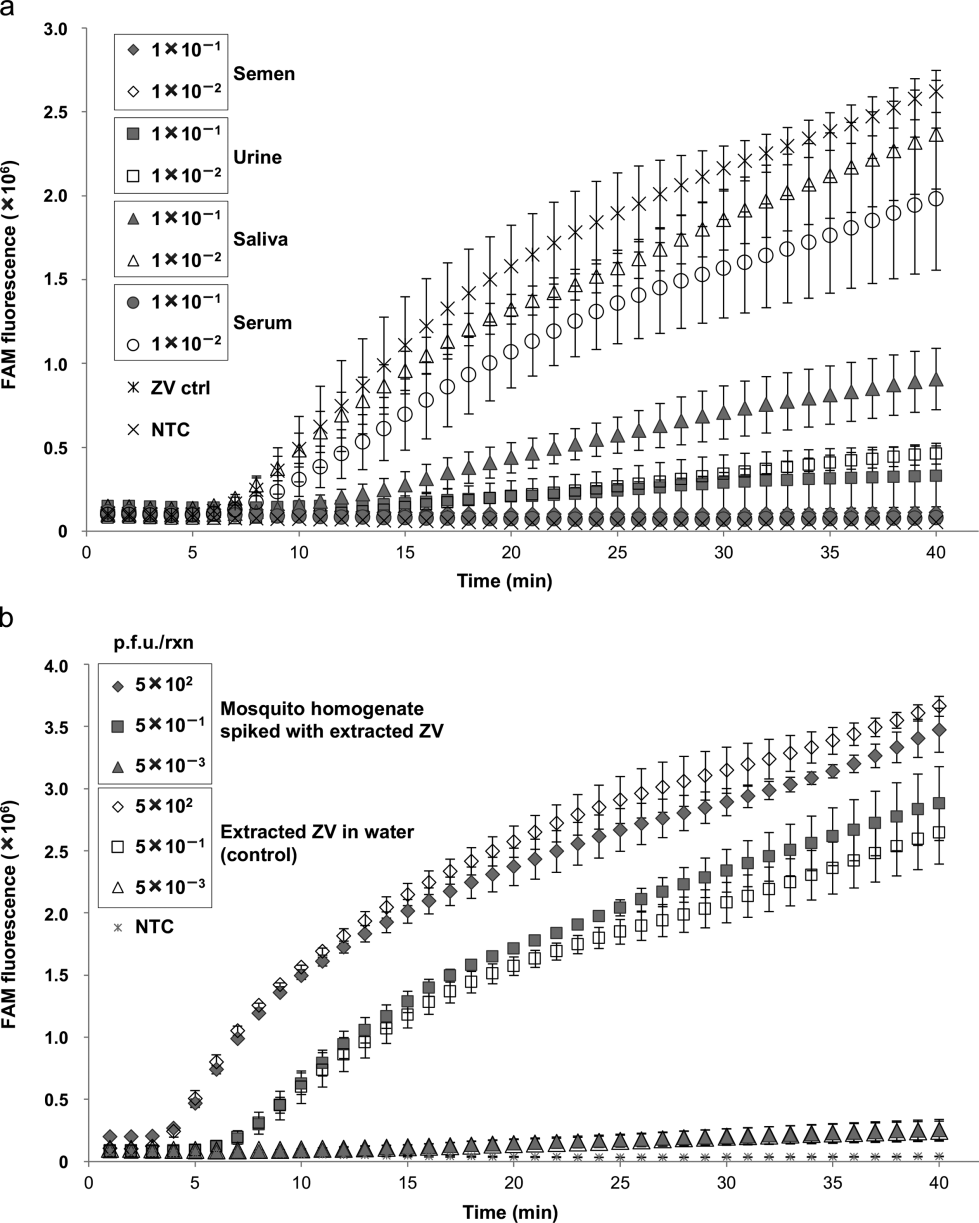
Effect of crude samples on the sensitivity of the ZIKV RT-RPA assay. (a) Inhibitory effect of semen, urine, saliva and serum samples diluted 10-fold and 100-fold on the ability of the ZIKV RT-RPA assay to detect extracted nucleic acid from cultured Zika virus (South American strain – KU501215) used at 1×10^2^ p.f.u. per reaction in each sample. The fluorescence signals from these are compared to the signal generated from the amplification of 1×10^2^ p.f.u. per reaction of the same extracted virus in nuclease-free water and the non-template control (NTC). (b) Inhibitory effect of homogenized mosquito preparations on the performance of the ZIKV RT-RPA assay. Crude neat homogenized pooled mosquito samples were spiked with extracted nucleic acid corresponding to 5×10^2^ p.f.u. from cultured ZIKV (South American strain – KU501215) and diluted 1000-fold and 100 000-fold. Amplification of these in the ZIKV RT-RPA assay was compared to the signal generated for the same dilutions of viral nucleic acid in nuclease-free water and the non-template control (NTC). Amplification curves show the average total fluorescence values from three independent ZIKV RT-RPA assays; standard deviations are represented as error bars.

To determine the capability of the ZIKV RT-RPA assay to tolerate the inhibitors present in crude samples, negative pooled neat donor semen, urine, saliva and serum samples were diluted 10-fold and 100-fold and spiked with extracted nucleic acid from South American cultured ZIKV (strain PRVABC59, NCBI accession number KU501215, from Puerto Rico, 2015) equivalent to 1×10^2^ p.f.u. per reaction to mimic crude clinical samples. The spiked crude samples were then analysed directly by the RT-RPA assay and compared to extracted virus RNA in nuclease-free water as a control (Fig. 4a). Positive results were detected from spiked samples and, in particular, robust detection was achieved in the spiked saliva samples. ZIKV nucleic acid was detected in both 10-fold- and 100-fold-diluted saliva samples, although the 10-fold-diluted sample was partially inhibitory to total fluorescence and TTP detection. The ZIKV RT-RPA assay efficiency for the 100-fold-diluted saliva sample was not significantly different from that of the control viral RNA (Fig. 4a). It was observed that semen was completely inhibitory to the ZIKV RT-RPA assay, whereas urine was partially inhibitory, with some amplification of the target region in the 10-fold-diluted sample, while the 100-fold-diluted urine sample was detected, but with reduced total fluorescence and delayed TTP when compared to the control virus extract in water (Fig. 4a). Serum needed to be diluted 100-fold to be detected successfully (Fig. 4a).

#### Mosquito samples

To investigate the applicability of the ZIKV RT-RPA assay as a potential in-field surveillance tool to monitor infected mosquito populations, mosquitoes were homogenized in nuclease-free water and supernatant from crude homogenate was spiked with extracted nucleic acid from the South American cultured ZIKV (strain PRVABC59, NCBI accession number KU501215, from Puerto Rico, 2015) equivalent to 5×10^2^ p.f.u. to mimic crude samples. The spiked neat crude sample was then diluted 1000-fold and 100 000-fold and analysed using the ZIKV RT-RPA assay and compared to control extracted ZIKV RNA in water at the same dilutions (Fig. 4b). No significant inhibition of target detection by the mosquito homogenate was observed in any of the dilutions tested. There was no difference in the TTP signal and total fluorescence between the viral RNA in the neat mosquito homogenate and the nuclease-free water. The same results were observed for the 1000-fold and 100 000-fold diluted samples, suggesting that the ZIKV RT-RPA may be used as a surveillance tool on crude mosquito samples (Fig. 4b).

### ZIKV RT-RPA assay can be performed on a portable battery-operated device

**Fig. 5.**
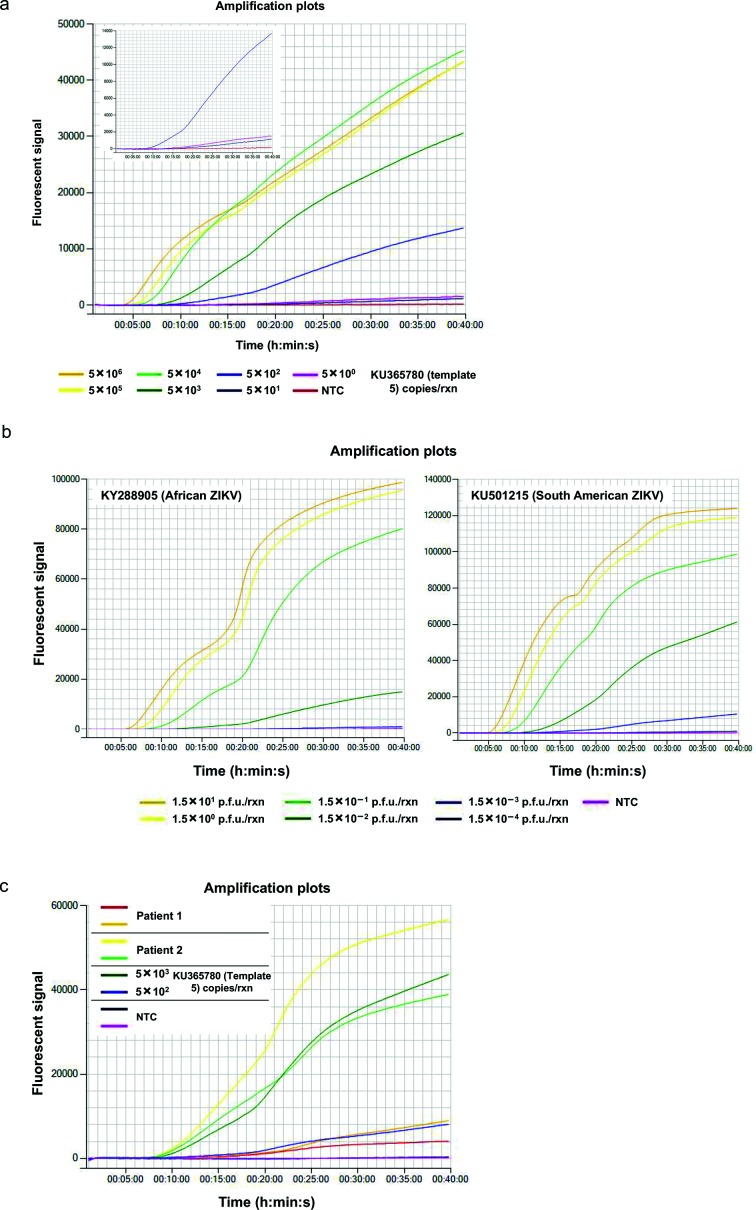
Performance of the ZIKV RT-RPA assay on a portable battery-operated instrument, the Genie III by OptiGene. (a) Tenfold dilution series of Zika synthetic RNA template 5 (KU365780) used to determine the lowest number of target molecules detected by the ZIKV RT-RPA assay. The insert displays the fluorescent signal generated from the amplification of 500, 50 and 5 copies of ZIKV synthetic RNA template 5 in comparison to the non-template control (NTC) in the ZIKV RT-RPA assay. (b) Tenfold dilution series of extracted nucleic acid from two strains of cultured ZIKV (African – KY288905, left panel, and South American – KU501215, right panel), as detected by the ZIKV RT-RPA assay and compared to the non-template control (NTC). (c) Detection of clinical samples from two patients in duplicates with low ZIKV titre (patient 1) and high ZIKV titre (patient 2) in comparison to 5×10^3^ and 5×10^2^ copies of Zika synthetic RNA template 5 (KU365780) and the non-template control (NTC) by the ZIKV RT-RPA.

The suitability of the ZIKV RT-RPA assay as a field diagnostic tool was demonstrated by testing it on a Genie III portable instrument manufactured by OptiGene [71]. Five copies of the synthetic RNA fragment 5 (KU365780) were successfully detected within 21 min (Fig. 5a), and 1.5×10^1^ p.f.u./reaction of ZIKV whole-genome nucleic acid extracted from cultured African (KY288905) and South American (KU501215) strains were detected within 6 and 5 min, respectively (Fig. 5b). Extracted RNA from clinical samples from two different patients was also tested in duplicate and successfully detected by the ZIKV RT-RPA using the Genie III instrument (Fig. 5c).

## Discussion

New research data have accumulated in the past year, with the link between ZIKV infection and sexual transmission being identified, and virus particles being detected in saliva and other bodily fluids. It has, therefore, become more urgent to deliver a rapid diagnosis to provide relevant public health information that can help protect vulnerable and at-risk individuals [39, 40, 72]. In addition, a field-friendly assay would enable infection control in low resource settings and aid in vector surveillance and management.

A recombinase polymerase amplification (RPA) assay for the rapid detection of ZIKV nucleic acid was successfully developed, achieving 100 % reliable detection (five out of five runs) for 500 copies of template within 10 min (Fig. 2). Remarkably, the assay positively identified as few as five copies of target sequence, (in three out of five runs) and results were obtained within 22.2 min (Fig. S3, Table S1). Therefore, the limit of detection of the ZIKV RT-RPA assay is estimated to be between 500 and 5 copies.

We demonstrated that the developed ZIKV RT-RPA assay is robust and capable of tolerating sequence variability in the region of interest, even when this occurs within the probe and primer binding sequence (Table 1). All five 1.8 kb ZIKV synthetic RNA fragments were amplified in the ZIKV RT-RPA assay with similar efficiency, indicating that it would detect the ZIKV strains from recent outbreaks (Figs 1 and 3a, Table 3). Moreover, the RPA assay was able to detect extracted nucleic acid from cultured virus derived from both a recent outbreak in South America (strain PRVABC59, NCBI accession number KU501215, from Puerto Rico, 2015) and a historic African strain (strain MP1751, NCBI accession number KY288905, from Uganda, 1962) [66, 68]. It is possible that the greater sequence variability of the African ZIKV strain in the target reverse primer and probe binding sequences when compared to the South American strain, whose sequence matched the assay design exactly, may account for the more efficient detection of the South American strain. In addition, the comparison of the target amplification was made based on p.f.u.s per reaction and this does not provide an accurate measure of the number of copies of nucleic acid molecules. These may have varied widely between the two strains in an unknown fashion, since a single p.f.u. could equate to 200–1000 viral nucleic acid copies, depending on the viral strain and culturing conditions [7, 73–77]. Therefore, the nucleic acid equivalent copies for the two ZIKV strains used may have been between 3×10^3^ and 1.5×10^4^ of viral RNA molecules.

Sequence variability and target copy number may help explain the observed 83 % sensitivity for the ZIKV RT-RPA assay when compared to the RT-PCR assay in real samples, even though the ZIKV RT-RPA assay limit of detection was lower than that of the RT-PCR assay (Table 4, Fig. 2). Sequence variation in a critical location, such as the exonuclease recognition site, may cause a lack of or reduced detection of the target viral RNA when copy numbers are at the limit of detection of the RT-RPA assay. In addition, 5 and 50 copies of synthetic fragment were detected in 60 % of the runs, whereas it was only possible to test clinical samples once. Repeat testing may identify the negative samples as positive in the ZIKV RT-RPA assay, but unfortunately this was not possible due to the limited amount of samples available. The samples identified as negative by the RT-RPA, but positive by RT-PCR had very late Ct values and were barely above the cut-off point for that assay. All samples were stored at −20 ˚C for over a year before testing and were derived from either urine or semen, which may have adversely affected the stability and integrity of the ZIKV nucleic acid, as has been shown previously [78]. Therefore, due to the larger footprint of the ZIKV RT-RPA compared to the RT-PCR, sample degradation could have caused loss of the target amplification region and reduced the efficiency of detection by the ZIKV RT-RPA, but not the RT-PCR. Testing the clinical samples when fresh may further improve the efficiency of the RT-RPA assay. Nevertheless, the ZIKV RT-RPA was demonstrated to be 100 % specific, based on the viruses tested, as clinical samples identified to be positive for dengue and chikungunya were not detected by the ZIKV RT-RPA assay (Table S2).

The specificity of the ZIKV RT-RPA assay was further confirmed using a panel of extracted viral RNA from a range of closely related or clinically relevant samples, such as dengue 1–4, chikungunya and Spondweni viruses (Table 5). The ZIKV RT-RPA assay was shown to give negative results for all of the tested viral extracts, apart from highly concentrated Spondweni viral RNA. Both ZIKV and Spondweni virus are endemic to central Africa, but ZIKV has a considerably wider geographical distribution, which may aid differential diagnosis [79]. The two viruses are often confused with each other in clinical and serological diagnostic methods, although they may be distinguished by nucleic acid analysis [79]. While a highly concentrated sample of Spondweni virus RNA (Ct 22 by real-time PCR analysis) was weakly detected by the ZIKV RT-RPA assay, a 10-fold dilution of this sample eliminated the signal in the ZIKV RT-RPA analysis (Fig. S4). While Spondweni may in the future spread out of Africa and overlap the distribution of ZIKV, it is expected that in clinical samples, as for ZIKV, Spondweni viral RNA would not be present at sufficiently high copy numbers to be detected by the ZIKV RT-RPA assay [80].

The ZIKV RT-RPA assay was developed as a point-of-care in-field assay for rapid diagnosis in low resource settings. With this in mind, the inhibitory effect of various crude biological fluids on the ability of the RPA assay to detect ZIKV nucleic acid was investigated (Fig. 4a). The ZIKV RT-RPA assay was partially inhibited by serum and urine, whereas successful amplification of the target sequence occurred in all saliva samples. As ZIKV particles have been identified in saliva, this may provide the necessary medium for crude sample testing with minimal processing and specialist equipment [72]. Use of saliva would make the ZIKV RT-RPA assay more field-appropriate, as the collection of saliva samples is neither invasive, nor gender-specific. However, further investigation would be required to determine whether ZIKV titres in saliva are sufficiently high to render sample extraction and concentration unnecessary, as well as to determine the most appropriate window of opportunity for sample collection post symptom onset.

The versatility of the RPA assay could also be applied as a monitoring tool for infected vectors; mosquitoes could be captured and tested on-site without the need for a laboratory setting. It was observed that the crude mosquito extracts did not inhibit the detection of ZIKV RNA in any of the dilutions or the neat crude homogenate. Although in this study the mosquitoes were homogenized using specialist equipment, there are a number of commercially available kits adapted for the testing of other arthropods, such as ticks, for the presence of *Borellia*, for example, the ‘Care Plus Tick-Test – Lyme borreliose’ kit. These have been designed to liberate the pathogen from the vector by crushing the tick in a liquid medium supplied in the kit. Therefore, it would be possible to adopt a similar and simple procedure for extracting viral nucleic acid from mosquitoes, minimizing the need for complicated equipment and methods. Further work would be necessary to establish the optimum mosquito manipulations and sample preparation conditions.

The utility of the ZIKV RT-RPA as a field diagnostic tool was further demonstrated by its performance on a battery-operated portable instrument, the Genie III by OptiGene. Comparable results in terms of TTP detection were obtained from the portable device as for the real-time PCR machine, for synthetic RNA fragments, ZIKV whole-genome extracted nucleic acid and clinical samples, suggesting that the ZIKV RT-RPA assay would be suitable for an outbreak situation in a low resource setting.

The performance of the ZIKV RT-RPA assay developed in this study compares well with that of a previously published RT-RPA assay for the detection of ZIKV nucleic acid extracted from urine samples [81]. However, the current study augments the area of field-appropriate ZIKV diagnostic assays by examining the utility of the new ZIKV RT-RPA in the detection of viral nucleic acid derived from different clinical samples, as well as target amplification using various ZIKV strains and crude sample preparations.

The developed ZIKV RT-RPA assay was shown to be highly sensitive, specific and versatile as a point-of-care diagnostic assay, as well as a valuable surveillance tool. With continuing developments in the area of isothermal assays, there is potential for their greater adaptation and suitability for field-testing when compared to traditional nucleic acid diagnostic methods, such as RT-PCR. The RPA assay, with its lower energy requirements, can be performed on a light, portable battery-operated device with minimal to no sample preparation, making it suitable for a low resource setting, basic laboratory set-up and non-specialist personnel. It could therefore shape the future of simple and rapid diagnostic methods.

## Methods

### Primer, probe and template design

The target area for the development of the ZIKV RT-RPA assay was selected based on the genome alignments of publicly available ZIKV genomes. Seven ZIKV genome sequences, derived from clinical isolates of outbreaks between 2007 and 2015, were selected and aligned to confirm the area of greatest sequence conservation. A set of forward and reverse primers and probes were designed within the envelope protein E gene sequence and tested in all possible combinations. The most favourable selection was verified to be specific for ZIKV using NCBI blast and was advanced for further validation.

The synthetic template fragments were designed as follows: template 1 (NCBI accession number EU545988), 1048–2873 bp; template 2 (NCBI accession number KJ776791), 1095–2920 bp, template 3 (NCBI accession number KU321639), 1153–2978 bp; template 4 (NCBI accession number KU365778), 1142–2967 bp, template 5 (NCBI accession number KU365780), 1141–2966 bp [14, 64, 65, 67]. Each fragment was designed with a T7 promoter sequence at the 5′-end and an SP6 promoter sequence at the 3′-end (Fig. S1).

Sequence analysis and alignments were performed using SeqBuilder, MegAlign and MegAlign Pro software (DNASTAR Lasergene 14 package).

### Primer, probe and template preparation

The selected forward and reverse primers were obtained from Integrated DNA Technologies at HPLC grade purification. The chosen probe was procured from ATDBio at double HPLC purification grade. The primers and probe were purchased in a lyophilized form and resuspended in TE buffer (10 mM Tris-HCl, 1 mM disodium EDTA, pH 8.0, BioUltra, Sigma) at 100 µM concentration. Working solutions were prepared at 10 µM concentrations in nuclease-free water (Millipore) and used at the appropriate concentrations.

Synthetic template fragments were purchased as 1800-bp lyophilized DNA gBlocks from Integrated DNA Technologies (Fig. S1). These were then briefly centrifuged at 3000 ***g***, resuspended in TE buffer at 10 ng µl^−1^ concentration and incubated at 50 ˚C for 20 min. gBlocks (200 pg per reaction) were amplified using Q5 Hot Start High-Fidelity 2X Master Mix (NEB) and 0.5 µM of T7 tail Fw and SP6 tail Rev primers according to the manufacturer’s instructions, using the following cycling conditions: denaturation at 98 ˚C for 30 s, followed by the amplification stage consisting of 35 cycles of denaturation at 98 ˚C for 10 s, primer annealing at 65 ˚C for 30 s and an extension at 72 ˚C for 1 min. The generated fragments were resolved on a 1 % agarose gel and gel-extracted using a QIAquick Gel Extraction kit (QIAgen). RNA template fragments were synthesized from the amplified and purified gBlock DNA fragments at approximately 1 µg per reaction using the HiScribe T7 High Yield RNA Synthesis kit (NEB) at 37 ˚C for 2 h according to the manufacturer’s instructions. The DNA template was then removed from the RNA samples by adding 4 units of DNase I (RNase-free, NEB) per sample and incubating at 37 ˚C for 15 min according to the manufacturer’s recommendations. Synthetic RNA fragments were subsequently purified using an RNeasy Mini kit (QIAgen). Synthetic RNA template fragments were quantified on a Qubit 3.0 Fluorometer using a Qubit RNA BR Assay kit (Thermo Fisher Scientific). RNA quality was assessed on a 2100 Bioanalyzer Instrument (Agilent) using an RNA 6000 Nano kit (Agilent).

### Zika nucleic acid detection

The RPA assay to detect the ZIKV target was performed in a 50 µl reaction volume using the TwistAmp exo RT kit (TwistDx, Cambridge, UK). The reaction composition was as follows: 2.1 µl of 10 µM Zika RPA Fw, 4.2 µl of 10 µM Zika RPA Rev, 0.6 µl of 10 µM Zika RPA probe, 29.5 µl of rehydration buffer, 3.6 µl of nuclease-free water and 5 µl of template. The reaction mix was added to the TwistAmp exo RT kit pellet and mixed gently to give a homogeneous suspension. Magnesium acetate, supplied at 280 mM concentration, was diluted twofold with nuclease-free water to a working concentration of 140 mM, from which 5 µl were added into each reaction. The ZIKV RT-RPA was performed at 41 ˚C for 40 min on a QuantStudio Flex 7 real-time PCR machine (Thermo Fisher Scientific) with fluorescence detection every 60 s in the FAM channel, without ROX passive reference. The threshold was set at 50 000 Rn.

The real-time PCR assay used to detect ZIKV targets was adapted from Lanciotti *et al.* [14]. Briefly, each reaction was performed using 0.9 µM of ZIKV 1086 forward primer, 0.9 µM of ZIKV 1162c reverse primer, 1 µM of ZIKV 1107-FAM probe, 0.8 µl of SuperScript III *Taq* and 10 µl of 2× reaction buffer from a SuperScript III Platinum One-Step qRT-PCR kit (Thermo Fisher Scientific), 0.5 mM MgSO_4_, 5 µl of template and a sufficient volume of nuclease-free water to achieve 20 µl total volume. The reverse transcriptase step was performed at 50 ˚C for 10 min, followed by denaturation at 95 ˚C for 2 min and an amplification stage consisting of 45 cycles of denaturation at 95 ˚C for 10 s and annealing/extension at 60 ˚C for 40 min. Fluorescence was detected in the FAM channel during the extension step of each cycle without ROX passive reference and the threshold was set at 1 000 000 ΔRn.

### Viral sample preparation

Two ZIKV strains, African ZIKV (strain MP1751, NCBI accession number KY288905, from Uganda, 1962) and South American ZIKV (strain PRVABC59, NCBI accession number KU501215, from Puerto Rico, 2015) were cultured, and viral RNA was extracted using a QIAmp viral RNA kit (QIAgen) [66, 68].

Zika viral nucleic acid was extracted from a selection of clinical samples using either a QIAmp viral RNA kit (QIAgen) or an EZ1 automated extraction platform (QIAgen), as specified by the manufacturer’s protocols.

Dengue 1 virus (strain Hawaii A, NCBI accession number KM204119), dengue 2 virus (strain R062, NCBI accession number NC_001474), dengue 3 virus (strain TC3), dengue 4 virus (strain TC25), West Nile virus (strain NY99, NCBI accession number AF196835), Mayaro virus (strain TC652, NCBI accession number NC_003417), yellow fever virus (strain FNT) and Rift Valley fever virus (strain h85/09) extracted RNA were obtained from Culture Collections, Public Health England [82–85].

St Louis encephalitis virus (strain MSI-7, NCBI accession number DQ359217) and La Crosse virus (EVAg stocks, NCBI accession number NC_004108) were cultured and viral RNA was extracted using QIAmp viral RNA kits (QIAgen) [86, 87].

Powassan virus, Usutu virus, O'nyong'nyong virus (strain Ang'mom), Karshi virus (strain 30517, NCBI accession numbers EU303204 and EU073997), Oropouche virus (EVAg stocks) and Spondweni virus (strain SM-6 V-1s, NCBI accession number DQ859064) RNA had been previously extracted and tested using the ZIKV RT-RPA assay [88–90]. Extracted nucleic acid samples from all selected viruses were tested using specific real-time PCR assays and the Ct values were determined to range between 20 and 30.

### Clinical samples analysis

A selection of 55 clinical samples were received for routine diagnostics as part of the reference work of the Rare and Imported Pathogens Laboratory (PHE Porton) and analysed using the ZIKV RT-RPA assay and the ZIKV real-time PCR assay. These included 8 semen, 35 urine, 5 serum, 2 whole blood and 5 samples defined as ‘other clinical’. Ethical approval for further studies using samples for the improvement of diagnostic assays without the requirement of informed consent from patients was obtained from the PHE Ethics Committee in Research.

### Crude sample preparation

Semen (pooled human donors), urine (pooled human donors) and saliva (pooled human donors) were purchased from Lee Biosolutions, Inc. Serum (from eight donors) was collected from volunteers at Public Health England and pooled. Serum collection was performed under the ethical approval of the Public Health England Research Ethics and Governance Group. Semen, urine, saliva and serum samples were diluted 10-fold and 100-fold and each sample was spiked with extracted cultured ZIKV nucleic acid (South American Zika virus, strain PRVABC59, NCBI accession number KU501215, from Puerto Rico, 2015), equivalent to 1×10^2^ p.f.u. [66]. Control extracted virus nucleic acid resuspended in nuclease-free water was used as a reference sample. Samples were analysed in duplicate using the ZIKV RT-RPA assay.

Wild mosquitoes (*Ochlerotatus detritus*, also known as *Aedes detritus*) were collected at Dee March, Merseyside, UK, on 30 September 2016. Each mosquito was homogenized using a Precellys tissue homogenizer in 300 µl nuclease-free water using CK28-R 2 ml reinforced tubes for 2×20 s at 4000 r.p.m., with 30 s breaks. Homogenized samples were centrifuged at 8000 r.p.m. for 5 min and cleared supernatant was removed to a fresh nuclease-free microcentrifuge tube. Mosquito homogenates were then pooled and aliquoted out for individual use. A neat homogenized and pooled mosquito sample was spiked with extracted cultured Zika virus nucleic acid (South American Zika virus, strain PRVABC59, NCBI accession number KU501215, from Puerto Rico, 2015) equivalent to 5×10^2^ p.f.u. [66]. The neat crude sample was then diluted 1000-fold and 100 000-fold using nuclease-free water and compared to extracted virus in water at the same dilutions. Samples were analysed using the ZIKV RT-RPA assay in duplicate. Spiking of different aliquots was performed in triplicate to obtain independent repeats. Average fluorescent values were calculated and plotted as a function of time. Standard deviations from the three independent repeats were plotted as error bars.

## Supplementary Data

Supplementary File 1
