# Supplementary material for: Point-of-care diagnostic assay for the detection of Zika virus using the recombinase polymerase amplification method
Source: J Gen Virol. 2018 Jun 13;99(8):1012–26. doi: 10.1099/jgv.0.001083 (PMC6171711; doi:10.1099/jgv.0.001083)
Supplement: Supplementary File 1 [file jgv-99-1012-s001.pdf]

Supplementary material Figure and Table legends

Sup. Table 1

Time to positive (TTP) signal in the detection of low copy number of ZIKV synthetic RNA template 5 (from $5x10^{1}$ to $5x10^{-1}$ copies per reaction) and compared to the non-template control (NTC) using the ZIKV RT-RPA assay. For comparison, the time necessary to detect the target using the published Zika RT-PCR is also shown.

Sup. Table 2

Confirmation of the specificity of the ZIKV RT-RPA assay using a selection Dengue-positive and 4 Chikungunya-positive clinical samples. The Dengue-positive and Chikungunya-positive status of the samples was confirmed by Dengue-specific and Chikungunya-specific RT-PCR, respectively, with outcomes and Ct values indicated.

Sup. Fig. 1

Sequences of the synthetic fragments used in the ZIKV RPA assay development and ZIKV RPA amplified region within utilised extracted ZIKV genomes.

a. Synthetic fragments 1 (Template 1 – EU545988), 2 (Template 2 – KJ776791), 3 (Template 3 – KU321639), 4 (Template 4 – KU365778) and 5 (Template 5 – KU365780) sequences shown 5'→3' direction. Underlined within each sequence are the T7 promoter at the 5'-end and the SP6 promoter at the 3'-end, used to amplify and synthesize the synthetic RNA fragments.

b. Sequences of the ZIKV RPA region of the African ZIKV (Strain MP1751, KY288905, from Uganda, 1962) and the South American ZIKV (Strain PRVABC59, KU501215, from Puerto Rico, 2015)

Sup. Fig. 2

Agarose gel analysis of the sizes of fragments generated in the ZIKV RT-RPA assay using synthetic fragments (Templates 1-5) and extracted ZIKV nucleic acid (African and South American Zika virus strains). Nuclease-free water instead of nucleic acid was used as a non-template control (NTC). Relative fragment sizes were confirmed using the E-GelÆ Low Range Quantitative DNA Ladder and marker sizes are indicated on the gel image.

Sup. Fig. 3

ZIKV RT-RPA assay sensitivity and performance using low copy number of template in comparison to ZIKV RT-PCR. Fluorescent signal generated from the amplification of 50, 5 and 0.5 copies per reaction of Zika synthetic RNA template 5 (KU365780) and compared to the non-template control (NTC) using the ZIKV RT-RPA assay. Amplification curves show the average total fluorescence values from five independent ZIKV RT-RPA assays; standard deviations are represented as error bars.

Sup. Fig. 4

Detection of cultured extracted Spondweni virus nucleic acid using the ZIKV RT-RPA assay. Signal from dilutions of $1x10^{-2}$ and $1x10^{-4}$ of the neat extracted Spondweni viral RNA are compared to signal generated using $1x10^{-4}$ dilution of neat extracted ZIKV RNA. Nuclease-

free water used in place of viral nucleic acid represents the non-template control (NTC). Amplification curves show the average total fluorescence values from three independent ZIKV RT-RPA assays, standard deviations are represented as error bars.

Sup. Fig. 1

Template 1 (EU545988)

GCGCTAATACGACTCACTATAGGGTGCTATGAGGCATCAATATCGGACATGGCTTCGGACAGCCGCTGCCCAACACAAGGTGAAGCCTACCTTGACAAGCAGTCAGA
CACTCAATATGTCTGCAAAAGAACGTTAGTGGACAGAGGCTGGGGAAATGGATGTGGACTTTTTGGCAAAGGGAGCCTGGTGACATGCGCTAAGTTTGCATGCTCCAA
GAAAATGACCGGGAAGAGCATCCAGCCAGAGAATCTGGAGTACCGGATAATGCTGTCAGTTCATGGCTCCCAGCACAGTGGGATGATCGTTAATGACACAGGACATG
AAACTGATGAGAATAGAGCGAAGGTTGAGATAACGCCCAATTCACCAAGAGCTGAAGCCACCCTGGGGGGTTTTGGAAGCCTAGGACTTGATTGTGAACCGAGGACA
GGCCTTGACTTTTCAGATTTGTATTACTTGACTATGAATAACAAGCACTGGTTGGTTCACAAGGAGTGGTTCCACGACATTCCATTACCTTGGCATGCTGGGGCAGACA
CCGGAACTCCACATTGGAACAACAAAGAAGCATTGGTAGAGTTCAAGGACGCACATGCCAAAAGGCAAACTGTCGTGGTTCTAGGGAGTCAAGAAGGAGCAGTTCACA
CGGCCCTTGCTGGAGCTCTGGAGGCTGAGATGGATGGTGCAAAGGGAAGGCTGTCCTCTGGCCACTTGAAATGTCGCCTGAAAATGGATAAACTTAGATTGAAGGGC
GTGTCATACTCCTTGTGTACCGCAGCGTTCACATTCACCAAGATCCCGGCTGAAACACTGCACGGGACAGTCACAGTGGAGGTACAGTACGCAGGGACAGATGGACC
CTGCAAGGTTCCAGCTCAGATGGCGGTGGACATGCAAACTCTGACCCCAGTTGGGAGGCTGATAACCGCTAACCCTGTAATCACTGAAAGCACTGAGAACTCTAAGAT
GATGCTGGAACTTGATCCACCATTTGGGGACTCTTACATTGTCATAGGAGTCGGGGAGAAGAAGATCACCCATCACTGGCACAGGAGTGGCAGCACCATTGGAAAAGC
ATTTGAAGCCACTGTGAGAGGTGCCAAGAGAATGGCAGTCTTGGGAGACACAGCCTGGGATTTTGGATCAGTTGGAGGTGCTCTCAACTCATTGGGCAAGGGCATCC
ATCAAATTTTTGGAGCAGCTTTCAAATCATTGTTTGGAGGAATGTCCTGGTTCTCACAAATTCTCATTGGAACGTTGCTGGTGTGGTTGGGTCTGAATACAAAGAATGGA
TCTATTTCCCTTACGTGCTTGGCCTTAGGGGGAGTGTTGATCTTTTTATCCACAGCCGTCTCTGCTGATGTGGGGTGCTCGGTGGACTTCTCAAAGAAGGAAACGAGAT
GCGGTACGGGGGTGTTCGTCTATAACGACGTTGATGCCTGGAGGGACAGGTACAAGTACCATCCTGACTCCCCTCGTAGATTAGCAGCAGCAGTCAAGCAAGCCTGG
GAAGATGGGATCTGTGGGATCTCCTCTGTTTCAAGAATGGAAAACATCATGTGGAGATCAGTAGAAGGGGAGCTCAACGCAATCCTGGAAGAGAATGGAGTTCAACTG
ACGGTCGTTGTGGGATCTGTAAAAAACCCCATGTGGAGAGGTCCACAGAGATTGCCCGTGCCTGTGAACGAGCTGCCCCACGGCTGGAAGGCTTGGGGGAAATCGTA
CTTCGTCAGAGCAGCAAAGACAAATAACAGCTTTGTCGTGGATGGTGACACACTGAAGGAATGCCCACTCAAACATAGAGCATGGAACAGCTTTCTTGTGGAGGATCA
TGGGTTCGGGGTATTTCA**TTCTATAGTGTCACCTAAATGCGC**

Template 2 (KJ776791)

GCGCTAATACGACTCACTATAGGGTGCTATGAGGCATCAATATCGGACATGGCTTCGGACAGCCGCTGCCCAACACAAGGTGAAGCCTACCTTGACAAGCAATCAGA
CACTCAATATGTCTGCAAAAGAACGTTAGTGGACAGAGGCTGGGGAAATGGATGTGGACTTTTTGGCAAAGGGAGCCTGGTGACATGCGCTAAGTTTGCATGCTCCAA
GAAAATGACCGGGAAGAGCATCCAGCCAGAGAATCTGGAGTACCGGATAATGCTGTCAGTTCATGGCTCCCAGCACAGTGGGATGATCGTTAATGACACAGGACATG
AAACTGATGAGAATAGAGCGAAGGTTGAGATAACGCCCAATTCACCAAGAGCCGAAGCCACCCTGGGGGGTTTTGGAAGCCTAGGACTTGATTGTGAACCGAGGACA
GGCCTTGACTTTTCAGATTTGTATTACTTGACTATGAATAACAAGCACTGGTTGGTTCACAAGGAGTGGTTCCACGACATTCCATTACCTTGGCACGCTGGGGCAGACA
CCGGAACTCCACACTGGAACAACAAAGAAGCACTGGTAGAGTTCAAGGACGCACATGCCAAAAGGCAAACTGTCGTGGTTCTAGGGAGTCAAGAAGGAGCAGTTCAC
ACGGCCCTTGCTGGAGCTCTGGAGGCTGAGATGGATGGTGCAAAGGGAAGGCTGTCCTCTGGCCACTTGAAATGTCGCCTGAAAATGGATAAACTTAGATTGAAGGG
CGTGTCATACTCCTTGTGTACCGCAGCGTTCACATTCACCAAGATCCCGGCTGAAACACTGCACGGGACAGTCACAGTGGAGGTACAGTACGCAGGGACAGATGGAC
CTTGCAAGGTTCCAGCTCAGATGGCGGTGGACATGCAAACTCTGACCCCAGTTGGGAGGTTGATAACCGCTAACCCCGTAATCACTGAAAGCACTGAGAACTCTAAGA
TGATGCTGGAACTTGATCCACCATTTGGGGACTCTTACATTGTCATAGGAGTCGGGGAGAAGAAGATCACCCACCACTGGCACAGGAGTGGCAGCACCATTGGAAAAG
CATTTGAAGCCACTGTGAGAGGTGCCAAGAGAATGGCAGTCTTGGGAGACACAGCCTGGGACTTTGGATCAGTTGGAGGCGCTCTCAACTCATTGGGCAAGGGCATC
CATCAAATTTTTGGAGCAGCTTTCAAATCATTGTTTGGAGGAATGTCCTGGTTCTCACAAATTCTCATTGGAACGTTGCTGATGTGGTTGGGTCTGAACACAAAGAATGG
ATCTATTTCCCTTATGTGCTTGGCCTTAGGGGGAGTGTTGATCTTCTTATCCACAGCTGTCTCTGCTGATGTGGGGTGCTCGGTGGACTTCTCAAAGAAGGAGACGAGA
TGCGGTACAGGGGTGTTCGTCTATAACGACGTTGAAGCCTGGAGGGACAGGTACAAGTACCATCCTGACTCCCCCCGTAGATTGGCAGCAGCAGTCAAGCAAGCCTG
GGAAGATGGTATCTGTGGGATCTCCTCTGTTTCAAGAATGGAAAACATCATGTGGAGATCAGTAGAAGGGGAGCTCAACGCAATCCTGGAAGAGAATGGAGTTCAACT
GACGGTCGTTGTGGGATCTGTAAAAAACCCCATGTGGAGAGGTCCACAGAGATTGCCCGTGCCTGTGAACGAGCTGCCCCACGGCTGGAAGGCTTGGGGGAAATCG
TACTTCGTCAGAGCAGCAAAGACAAATAACAGCTTTGTCGTGGATGGTGACACACTGAAGGAATGCCCACTCAAACATAGAGCATGGAACAGCTTTCTTGTGGAGGAT
CATGGGTTCGGGGTATTTCA**TTCTATAGTGTCACCTAAATGCGC**

Template 3 (KU321639)

GCGCTAATACGACTCACTATAGGGTGCTATGAGGCATCAATATCAGACATGGCTTCGGACAGCCGCTGCCCAACACAAGGTGAAGCCTACCTTGACAAGCAATCAGA
CACTCAATATGTCTGCAAAAGAACGTTAGTGGACAGAGGCTGGGGAAATGGATGTGGACTTTTTGGCAAAGGGAGTCTGGTGACATGCGCTAAGTTTGCATGCTCCAA
GAAAATGACCGGGAAGAGCATCCAGCCAGAGAATCTGGAGTACCGGATAATGCTGTCAGTTCATGGCTCCCAGCACAGTGGGATGATCGTTAATGACACAGGACATG
AAACTGATGAGAATAGAGCGAAGGTTGAGATAACGCCCAATTCACCAAGAGCCGAAGCCACCCTGGGGGGTTTTGGAAGCCTAGGACTTGATTGTGAACCGAGGACA
GGCCTTGACTTTTCAGATTTGTATTACTTGACTATGAATAACAAGCACTGGTTGGTTCACAAGGAGTGGTTCCACGACATTCCATTACCTTGGCACGCTGGGGCAGACA
CCGGAACTCCACACTGGAACAACAAAGAAGCACTGGTAGAGTTCAAGGACGCACATGCCAAAAGGCAAACTGTCGTGGTTCTAGGGAGTCAAGAAGGAGCAGTTCAC
ACGGCCCTTGCTGGAGCTCTGGAGGCTGAGATGGATGGTGCAAAGGGAAGGCTGTCCTCTGGCCACTTGAAATGTCGCCTGAAAATGGATAAACTTAGATTGAAGGG
CGTGTCATACTCCTTGTGTACCGCAGCGTTCACATTCACCAAGATCCCGGCTGAAACACTGCACGGGACAGTCACAGTGGAGGTACAGTACGCAGGGACAGATGGAC
CTTGCAAGGTTCCAGCTCAGATGGCGGTGGACATGCAAACTCTGACCCCAGTTGGGAGGTTGATAACCGCTAACCCCGTAATCACTGAAAGCACTGAGAACTCTAAGA
TGATGCTGGAACTTGATCCACCATTTGGGGACTCTTACATTGTCATAGGAGTCGGGGAGAAGAAGATCACCCACCACTGGCACAGGAGTGGCAGCACCATTGGAAAAG
CATTTGAAGCCACTGTGAGAGGTGCCAAGAGAATGGCAGTCTTGGGAGACACAGCCTGGGACTTTGGATCAGTTGGAGGCGCTCTCAACTCATTGGGCAAGGGCATC
CATCAAATTTTTGGAGCAGCTTTCAAATCATTGTTTGGAGGAATGTCCTGGTTCTCACAAATTCTCATTGGAACGTTGCTGATGTGGTTGGGTCTGAACACAAAGAATGG
ATCTATTTCCCTTATGTGCTTGGCCTTAGGGGGAGTGTTGATCTTCTTATCCACAGCCGTCTCTGCTGATGTGGGGTGCTCGGTGGACTTCTCAAAGAAGGAGACGAG
ATGCGGTACAGGGGTGTTCGTCTATAACGACGTTGAAGCCTGGAGGGACAGGTACAAGTACCATCCTGACTCCCCCCGTAGATTGGCAGCAGCAGTCAAGCAAGCCT
GGGAAGATGGTATCTGCGGGATCTCCTCTGTTTCAAGAATGGAAAACATCATGTGGAGATCAGTAGAAGGGGAGCTCAACGCAATCCTGGAAGAGAATGGAGTTCAAC
TGACGGTCGTTGTGGGATCTGTAAAAAACCCCATGTGGAGAGGTCCACAGAGATTGCCCGTGCCTGTGAACGAGCTGCCCCACGGCTGGAAGGCTTGGGGGAAATC
GCACTTCGTCAGAGCAGCAAAGACAAATAACAGCTTTGTCGTGGATGGTGACACACTGAAGGAATGCCCACTCAAACATAGAGCATGGAACAGCTTTCTTGTGGAGGA
TCATGGGTTCGGGGTATTTCA**TTCTATAGTGTCACCTAAATGCGC**

Template 4 (KU365778)

GCGCTAATACGACTCACTATAGGGTGCTATGAGGCATCAATATCAGACATGGCTTCGGACAGCCGCTGCCCAACACAAGGTGAAGCCTACCTTGACAAGCAATCAGA
CACTCAATATGTCTGCAAAAGAACGTTAGTGGACAGAGGCTGGGGAAATGGATGTGGACTTTTTGGCAAAGGGAGCCTGGTGACATGCGCTAAGTTTGCATGCTCCAA
GAAAATGACCGGGAAGAGCATCCAGCCAGAGAATCTGGAGTACCGGATAATGCTGTCAGTTCATGGCTCCCAGCACAGTGGGATGATCGTTAATGACACAGGACATG
AAACTGATGAGAATAGAGCGAAAGTTGAGATAACGCCCAATTCACCAAGAGCCGAAGCCACCCTGGGGGGTTTTGGAAGCCTAGGACTTGATTGTGAACCGAGGACA
GGCCTTGACTTTTCAGATTTGTATTACTTGACTATGAATAACAAGCACTGGTTGGTTCACAAGGAGTGGTTCCACGACATTCCATTACCTTGGCACGCTGGGGCAGACA
CCGGAACTCCACACTGGAACAACAAAGAAGCACTGGTAGAGTTCAAGGACGCACATGCCAAAAGGCAAACTGTCGTGGTTCTAGGGAGTCAAGAAGGAGCAGTTCAC
ACGGCCCTTGCTGGAGCTCTGGAGGCTGAGATGGATGGTGCAAAGGGAAGGCTGTCCTCTGGCCACTTGAAATGTCGCCTGAAAATGGATAAACTTAGATTGAAGGG
CGTGTCATACTCCTTGTGTACTGCAGCGTTCACATTCACCAAGATCCCGGCTGAAACACTGCACGGGACAGTCACAGTGGAGGTACAGTACGCAGGGACAGATGGAC
CTTGCAAGGTTCCAGCTCAGATGGCGGTGGACATGCAAACTCTGACCCCAGTTGGGAGGTTGATAACCGCTAACCCCGTAATCACTGAAAGCACTGAGAACTCTAAGA
TGATGCTGGAACTTGATCCACCATTTGGGGACTCTTACATTGTCATAGGAGTCGGGGAGAAGAAGATCACCCACCACTGGCACAGGAGTGGCAGCACCATTGGAAAAG
CATTTGAAGCCACTGTGAGAGGTGCCAAGAGAATGGCAGTCTTGGGAGACACAGCCTGGGACTTTGGATCAGTTGGAGGCGCTCTCAACTCATTGGGCAAGGGCATC
CATCAAATTTTTGGAGCAGCTTTCAAATCATTGTTTGGAGGAATGTCCTGGTTCTCACAAATTCTCATTGGAACGTTGCTGATGTGGTTGGGTCTGAACACAAAGAATGG
ATCTATTTCCCTTATGTGCTTGGCCTTAGGGGGGGTGTTGATCTTCTTATCCACAGCCGTCTCTGCTGATGTGGGGTGCTCGGTGGACTTCTCAAAGAAGGAGACGAG
ATGCGGTACAGGGGTGTTCGTCTATAACGACGTTGAAGCCTGGAGGGACAGGTACAAGTACCATCCTGACTCCCCCCGTAGATTGGCAGCAGCAGTCAAGCAAGCCT
GGGAAGATGGTATCTGCGGGATCTCCTCTGTTTCAAGAATGGAAAACATCATGTGGAGATCAGTAGAAGGGGAGCTCAACGCAATCCTGGAAGAGAATGGAGTTCAAC
TGACGGTCGTTGTGGGATCTGTAAAAAACCCCATGTGGAGAGGTCCACAGAGATTGCCCGTGCCTGTGAACGAGCTGCCCCACGGCTGGAAGGCTTGGGGGAAATC
GTACTTCGTCAGAGCAGCAAAGACAAATAACAGCTTTGTCGTGGATGGTGACACACTGAAGGAATGCCCACTCAAACATAGAGCATGGAACAGCTTTCTTGTGGAGGA
TCATGGGTTCGGGGTATTTCA**TTCTATAGTGTCACCTAAATGCGC**

Template 5 (KU365780)

GCGCTAATACGACTCACTATAGGGTGCTATGAGGCATCAATATCAGACATGGCTTCGGACAGCCGCTGCCCAACACAAGGTGAAGCCTACCTTGACAAGCAATCAGA
CACTCAATATGTCTGCAAAAGAACGTTAGTGGACAGAGGCTGGGGAAATGGATGTGGACTTTTTGGCAAAGGGAGCCTGGTGACATGCGCTAAGTTTGCATGCTCCAA
GAAAATGACCGGGAAGAGCATCCAGCCAGAGAATCTGGAGTACCGGATAATGCTGTCAGTTCATGGCTCCCAGCACAGTGGGATGATTGTTAATGACACAGGACATGA
AACTGATGAGAATAGAGCGAAAGTTGAGATAACGCCCAATTCACCAAGAGCCGAAGCCACCCTGGGGGGTTTTGGAAGCCTAGGACTTGATTGTGAACCGAGGACAG
GCCTTGACTTTTCAGATTTGTATTACTTGACTATGAATAACAAGCACTGGTTGGTTCACAAGGAGTGGTTCCACGACATTCCATTACCTTGGCACGCTGGGGCAGACAC
CGGAACTCCACACTGGAACAACAAAGAAGCACTGGTAGAGTTCAAGGACGCACATGCCAAAAGGCAAACTGTCGTGGTTCTAGGGAGTCAAGAAGGAGCAGTTCACA
CGGCCCTTGCTGGAGCTCTGGAGGCTGAGATGGATGGTGCAAAGGGAAGGCTGTCCTCTGGCCACTTGAAATGTCGCCTGAAAATGGATAAACTTAGATTGAAGGGC
GTGTCATACTCCTTGTGTACTGCAGCGTTCACATTCACCAAGATCCCGGCTGAAACACTGCACGGGACAGTCACAGTGGAGGTACAGTACGCAGGGACAGATGGACC
TTGCAAGGTTCCAGCTCAGATGGCGGTGGACATGCAAACTCTGACCCCAGTTGGGAGGTTGATAACCGCTAACCCCGTAATCACTGAAAGCACTGAGAACTCTAAGAT
GATGCTGGAACTTGATCCACCATTTGGGGACTCTTACATTGTCATAGGAGTCGGGGAGAAGAAGATCACCCACCACTGGCACAGGAGTGGCAGCACCATTGGAAAAG
CATTTGAAGCCACTGTGAGAGGTGCCAAGAGAATGGCAGTCTTGGGAGACACAGCCTGGGACTTTGGATCAGTTGGAGGCGCTCTCAACTCATTGGGCAAGGGCATC
CATCAAATTTTTGGAGCAGCTTTCAAATCATTGTTTGGAGGAATGTCCTGGTTCTCACAAATTCTCATTGGAACGTTGCTGATGTGGTTGGGTCTGAACACAAAGAATGG
ATCTATTTCCCTTATGTGCTTGGCCTTAGGGGGAGTGTTGATCTTCTTATCCACAGCCGTCTCTGCTGATGTGGGGTGCTCGGTGGACTTCTCAAAGAAGGAGACGAG
ATGCGGTACAGGGGTGTTCGTCTATAACGACGTTGAAGCCTGGAGGGACAGGTACAAGTACCATCCTGACTCCCCCCGTAGATTGGCAGCAGCAGTCAAGCAAGCCT
GGGAAGATGGTATCTGCGGGATCTCCTCTGTTTCAAGAATGGAAAACATCATGTGGAGATCAGTAGAAGGGGAGCTCAACGCAATCCTGGAAGAGAATGGAGTTCAAC
TGACGGTCGTTGTGGGATCTGTAAAAAACCCCATGTGGAGAGGTCCACAGAGATTGCCCGTGCCTGTGAACGAGCTGCCCCACGGCTGGAAGGCTTGGGGGAAATC
GTACTTCGTCAGAGCAGCAAAGACAAATAACAGCTTTGTCGTGGATGGTGACACACTGAAGGAATGCCCACTCAAACATAGAGCATGGAACAGCTTTCTTGTGGAGGA
TCATGGGTTCGGGGTATTTCA**TTCTATAGTGTCACCTAAATGCGC**

African Zika virus (Strain MP1751, KY288905, from Uganda, 1962) RPA target

CCAACACAAGGTGAAGCCTACCTTGACAAGCAATCAGACACTCAATATGTTTGCAAAAGAACATTGGTGGACAGAGGTTGGGGAAATGGGTGTGGACTCTTTGGCAAA
GGGAGTTTGGTGACATGTGCTAAGTTCACGTGCTCCAAGAAGATGACTGGGAAGAGCATTCAGCCGGAGAA

South American Zika virus (Strain PRVABC59, KU501215, from Puerto Rico, 2015) RPA target

CCAACACAAGGTGAAGCCTACCTTGACAAGCAATCAGACACTCAATATGTCTGCAAAAGAACGTTAGTGGACAGAGGCTGGGGAAATGGATGTGGACTTTTTGGCAAA
GGGAGCCTGGTGACATGCGCTAAGTTTGCATGCTCCAAGAAAATGACCGGGAAGAGCATCCAGCCAGAGAA

Sup. Fig. 2

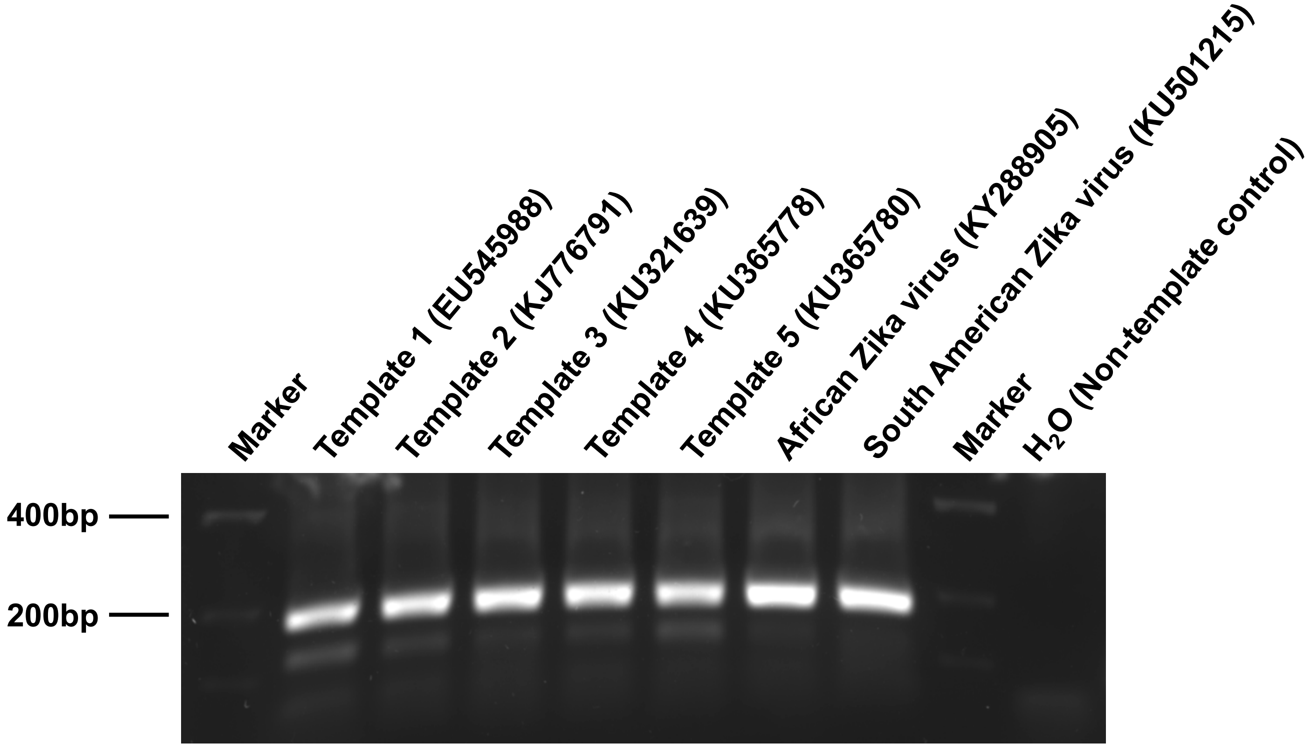
Marker
Template 1 (EU545988)
Template 2 (KJ776791)
Template 3 (KU321639)
Template 4 (KU365778)
Template 5 (KU365780)
African Zika virus (KY288905)
South American Zika virus (KU501215)
Marker
H_2O (Non-template control)
400bp
200bp

Sup. Fig. 3

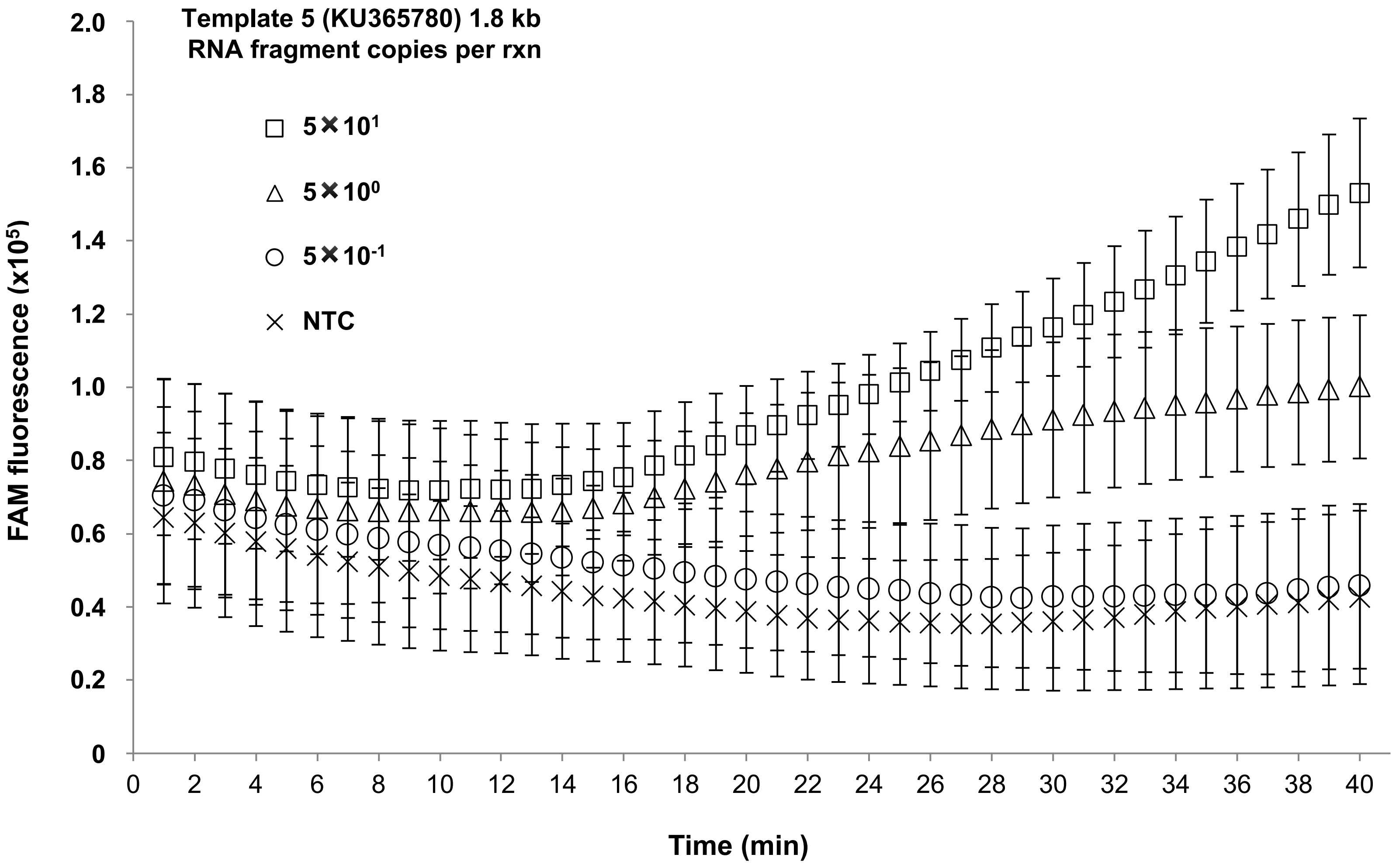

Template 5 (KU365780) 1.8 kb
RNA fragment copies per rxn
5×10^1
5×10^0
5×10^-1
NTC
FAM fluorescence (x10^5)
Time (min)
2.0
1.8
1.6
1.4
1.2
1.0
0.8
0.6
0.4
0.2
0
0 2 4 6 8 10 12 14 16 18 20 22 24 26 28 30 32 34 36 38 40

Sup. Table 1

Template 5 (KU365780) copies/rxn	RT-RPA TTP (mins)	RT-RPA result	RT-PCR TTP (mins)	RT-PCR result
$5x10^1$	21.73	+ (3/5)	60.33	+ (3/3)
$5x10^0$	22.20	+ (3/5)	Not detected	- (3/3)
$5x10^{-1}$	Not detected	- (5/5)	Not detected	- (3/3)
NTC	Not detected	- (5/5)	Not detected	- (3/3)

Sup. Fig. 4

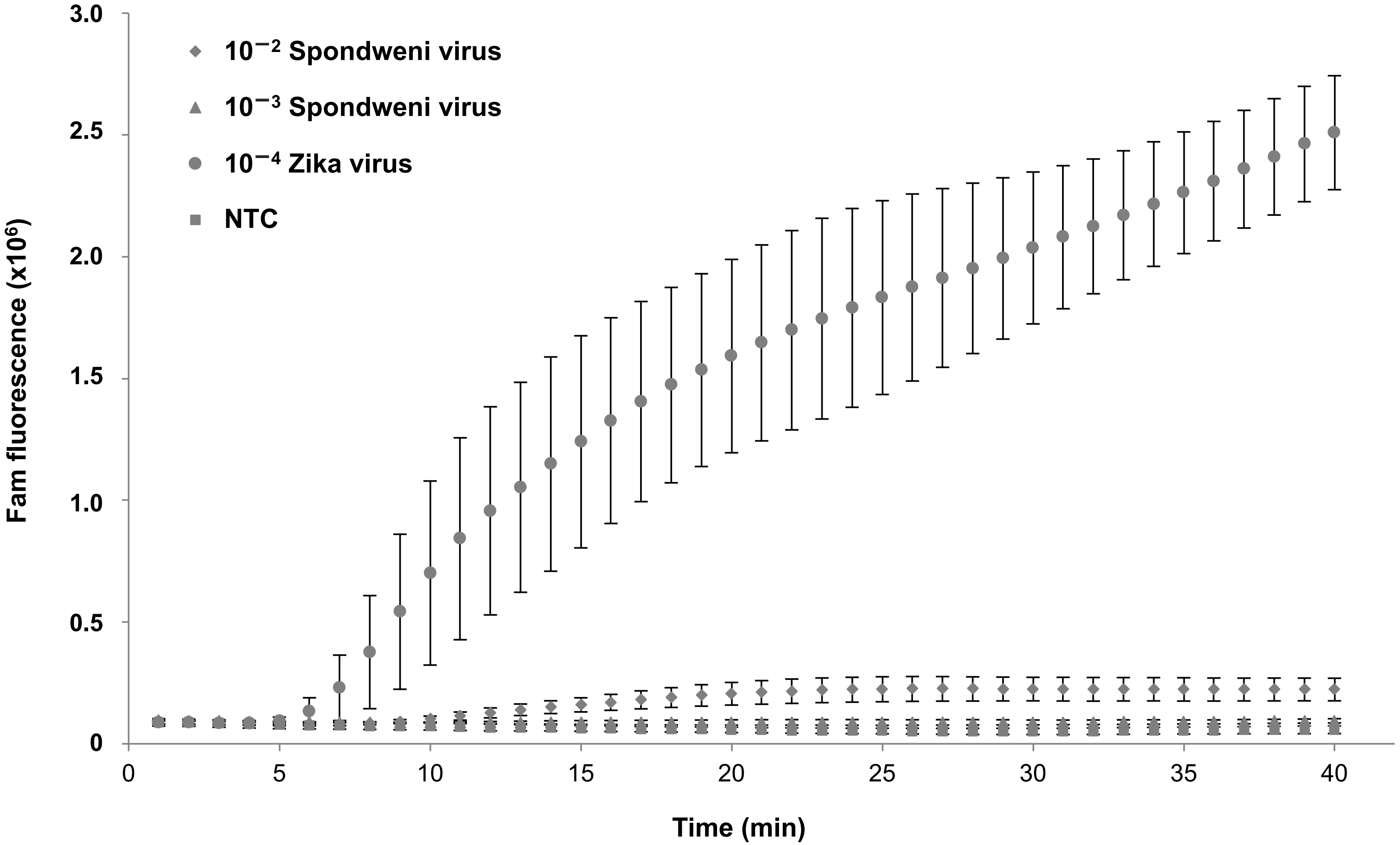

10^{-2} Spondweni virus
10^{-3} Spondweni virus
10^{-4} Zika virus
NTC
Fam fluorescence ($x10^6$)
3.0
2.5
2.0
1.5
1.0
0.5
0
0
5
10
15
20
25
30
35
40
Time (min)

Sup. Table 2

Sample No	Sample description	RT-RPA result	RT-PCR Ct	RT-PCR result
1	Dengue clinical	-	27.069	+
2	Dengue clinical	-	32.610	+
3	Dengue clinical	-	35.749	+
4	Dengue clinical	-	24.517	+
5	Dengue clinical	-	36.176	+
6	Dengue clinical	-	24.403	+
7	Dengue clinical	-	22.330	+
8	Dengue clinical	-	23.800	+
9	Dengue clinical	-	26.660	+
10	Dengue clinical	-	33.898	+
11	Dengue clinical	-	34.870	+
12	Chikungunya clinical	-	30.412	+
13	Chikungunya clinical	-	30.264	+
14	Chikungunya clinical	-	39.618	+
15	Chikungunya clinical	-	37.512	+
